# Intron–mRNA Matching Patterns in Differentially Expressed Genes in Cervical Cancer

**DOI:** 10.3390/biology15212004

**Published:** 2026-11-05

**Authors:** Pengzhe Sun, Yonge Feng, Zuwei Yan, Qiang Zhang

**Affiliations:** College of Science, Inner Mongolia Agricultural University, Hohhot 010018, China; sunpengzhe119@imau.edu.cn (P.S.); yefeng@imau.edu.cn (Y.F.)

**Keywords:** cervical cancer, differentially expressed genes, intron–mRNA matching, GC content, transcriptomics, sequence alignment

## Abstract

Cervical cancer (CC) is characterized by widespread transcriptomic dysregulation, yet sequence matching patterns between mRNAs and their corresponding introns remain insufficiently characterized in differentially expressed genes (DEGs). This study aimed to describe computationally predicted intron–mRNA matching features in the reference sequences of CC DEGs. DEGs were identified using Gene Expression Omnibus (GEO) and The Cancer Genome Atlas (TCGA) datasets, and the corresponding mRNA and intron sequences were retrieved from the Ensembl human reference genome. Reverse complementation combined with Smith–Waterman local alignment was used to analyze the positional distributions of predicted matching fragments across full-length mRNAs and within translation initiation and termination regions. Descriptive analyses were further stratified by the GC content of the matching fragments. On normalized full-length mRNA coordinates, high-GC fragments exhibited higher matching frequencies in certain segments near the 5′ end, whereas matching frequencies of low-GC fragments increased toward the 3′ end. Within the translation initiation region, predicted matching frequencies were generally higher upstream than downstream of the AUG codon, and the low-GC group exhibited a local peak near the initiation codon. Within the translation termination region, the low-GC group exhibited a pronounced peak downstream of the stop codon, whereas the high-GC group showed no similarly pronounced downstream peak and displayed greater positional fluctuations in matching frequency. Overall, the predicted matching distributions differed across the analyzed regions and GC groups. This study provides a sequence-level description of intron–mRNA matching patterns within this reference sequence collection of CC DEGs; their biological significance requires further validation.

## Simple Summary

Cervical cancer is associated with numerous changes in gene expression, yet the sequence relationships between messenger RNAs (mRNAs) and introns excised during RNA processing remain incompletely understood. This study selected genes reported to be differentially expressed in cervical cancer datasets and examined predicted sequence complementarity between their reference mRNA sequences and corresponding intron sequences in specific regions. Local alignment was used to identify the highest-scoring predicted complementary regions, and their positions were summarized across full-length mRNAs and around translation initiation and termination sites. Additional descriptive comparisons were performed between groups of matching fragments with different guanine and cytosine (GC) contents. The observed distribution patterns varied across transcript positions and between groups. These exploratory findings provide new descriptive insights at the sequence level.

## 1. Introduction

Introns, once regarded merely as non-functional byproducts of pre-mRNA splicing, are now recognized as important regulators of eukaryotic gene expression [1]. In addition to their roles in constitutive and alternative splicing, introns can influence transcription efficiency, nuclear export, and translation, thereby participating in multiple stages of RNA processing and post-transcriptional regulation [2]. They have also been implicated more broadly in genome evolution, proteomic diversification, and the maintenance of genomic stability [3]. These observations indicate that introns should not be viewed simply as intervening sequences, but rather as functionally relevant components of gene regulatory systems.

One important aspect of intron function is its potential interaction with complementary sequence regions within mRNA [4]. Such interactions may participate in the regulation of transcript processing, stability, and translation, and their disruption has been associated with aberrant splicing, dysregulation of non-coding RNA activity, and disease-related transcriptomic alterations [5,6,7]. In the broader context of cancer-associated RNA dysregulation, Oliaee et al. reviewed the roles of dysregulated microRNAs and their target genes in common spinal tumors, highlighting the relevance of non-coding RNA-mediated post-transcriptional regulation to tumor biology [8].

Zhang et al. analyzed the interaction potential between introns and mRNA using reverse complementary sequence matching and the Smith–Waterman local alignment method and systematically established the methodological framework. They defined the regions with the highest interaction probability between mRNA and the corresponding intronic sequences as the “optimal matching fragments” and comprehensively characterized the sequence features of these fragments as well as the distribution patterns of matching intensity along mRNA sequences [9,10,11].

The use of this sequence-analysis approach is motivated by previous computational studies of intron–mRNA matching features. Intron and exon sequences are initially transcribed as parts of a common pre-mRNA. Splicing can occur during or after transcription, releasing introns as lariat RNAs and joining the exons. Mature mRNA is generated upon completion of the relevant RNA-processing steps and is subsequently exported from the nucleus [12]. Excised introns generally undergo debranching and degradation, although some excised intron RNAs can persist [13,14]. Thus, a shared gene of origin does not establish temporal coexistence, subcellular colocalization, or physical interaction between an excised intron and its corresponding mature mRNA. In this study, local sequence alignment is used to characterize predicted sequence matches and potential complementarity. Previous studies by our group systematically characterized the cross-species conservation of intron–mRNA matching features. The research framework included multiple levels of datasets: genome-wide analyses of ribonucleoprotein gene sequences in *Caenorhabditis elegans*; local alignments between ribonucleoprotein RNA sequences and corresponding intronic sequences from 27 species, including humans, mice, rats, honeybees, and pufferfish [9,10]; and validation analyses using chromosome 1 genomic gene sequences from 13 species, including humans, mice, rats, dogs, chickens, cattle, zebrafish, nematodes, fruit flies, *Arabidopsis thaliana*, grapevine, rice, and poplar, as databases for mRNA–intron alignments [15]. These studies motivated the descriptive analysis of predicted intron–mRNA matching patterns in the present study.

Systematic studies on the evolutionary mechanisms of intron structure and length demonstrated coordinated evolution between intronic sequences and mRNA coding regions [11]. The hypothesis that intron–mRNA sequence matching corresponds to regulatory interactions has obtained computational support through a series of studies. Building upon the foundational work of Zhang et al. in identifying optimal matching fragments and characterizing matching distribution features, Bo et al. [15,16,17] and Cao et al. [18] further optimized the analytical methods and conducted extensive validations, confirming that complementary regions between introns and mRNAs are subject to evolutionary constraints and exhibit conserved distribution features across species [19]. Furthermore, these studies indicated that spliced introns may exert novel post-transcriptional regulatory functions through interactions with corresponding mRNAs across multiple species [15,16,17,18,19]. Collectively, these consistent findings strongly support the core hypothesis that intron–mRNA matching reflects potential regulatory interactions. On this basis, previous studies further suggested that low-GC predicted matching regions tend to occur more frequently within the 3′ untranslated region (3′UTR), whereas high-GC predicted regions are preferentially enriched within the 5′ untranslated region (5′UTR) [15,19]. In addition, complementary interactions are weaker in coding regions than in untranslated regions, highlighting the potential importance of regional sequence context in intron–mRNA matching [15,19].

Aberrant intronic or mRNA sequences that disrupt potential complementary regions may contribute to disease by altering normal regulatory processes. For example, intronic variants can induce abnormal splicing and impair tumor suppressor activity [20], whereas intron-associated interactions near untranslated regions may influence mRNA stability [21,22]. Introns have also been implicated in the regulation of oncogene-related splicing events [23,24]. Cancer biology is shaped by molecular alterations across multiple regulatory layers. Integrating genomic, transcriptomic, proteomic, and epigenomic information provides a broader framework for characterizing oncogenic determinants and tumor heterogeneity [25]. Within this framework, alterations in RNA-associated regulatory factors represent an additional aspect of cancer molecular characterization. For example, Valentine et al. characterized ADAR1 mutations and expression changes in human cancers and reported an association between reduced ADAR1 expression and increased BLCAP expression [26]. As ADAR1 participates in post-transcriptional RNA editing, this study illustrates the relevance of examining RNA regulatory factors alongside gene expression changes in cancer. Against this background, cervical cancer-related differentially expressed genes (DEGs) identified from cancer transcriptome datasets provide a relevant gene set for investigating computationally predicted intron–mRNA sequence complementarity using reference genome sequences. However, the matching features between these reference mRNAs and their corresponding introns remain insufficiently characterized, including variation observed after stratification by fragment GC content.

From a sequence-analysis perspective, local regions with high complementarity may represent informative sites for studying computationally predicted intron–mRNA potential complementarity. Systematic characterization of these regions may therefore help explore how sequence composition and transcript context jointly shape predicted matching features. In particular, examining the distribution of matching frequencies across full-length transcripts and around translation initiation and termination regions may provide a more detailed description of the positional features of such computationally predicted complementarity. This is especially relevant for cancer-related DEGs showing transcriptional dysregulation in tumor datasets.

In this study, we used GEO and TCGA transcriptomic datasets to identify genes differentially expressed in cervical cancer. The mRNA and intron sequences used for sequence analysis were retrieved from the Ensembl human reference genome rather than from tissue-specific transcript assemblies. We reverse-complemented the intron sequences and applied Smith–Waterman local alignment to characterize predicted matches with their corresponding reference mRNAs. We then examined the positional distributions of matching frequencies across full-length mRNAs and within translation initiation and termination regions, with additional descriptive stratification by the GC content of the matching fragments. By quantitatively describing these features, this study aimed to characterize the positional distributions and regional differences in predicted intron–mRNA matching within this DEG reference-sequence set, providing descriptive sequence-level information for subsequent evaluation using appropriate sequence controls and functional experiments.

## 2. Materials and Methods

### 2.1. Data Sources and Sample Collection

In this study, cervical cancer (CC) tissue expression data were collected from the Gene Expression Omnibus (GEO) and The Cancer Genome Atlas (TCGA) databases. The GEO datasets included GSE138080, GSE39001, GSE46857, GSE63514, and GSE63678, which were downloaded from the NCBI GEO database (National Center for Biotechnology Information, National Library of Medicine, Bethesda, MD, USA; https://www.ncbi.nlm.nih.gov/geo/ (accessed on 16 February 2025)). Based on the corresponding platform annotation files, probe-level expression matrices were converted into gene-level expression matrices, and the associated clinical information was extracted. After samples with incomplete clinical information were excluded, GSE138080 contained 20 samples, including 10 tumor samples and 10 normal samples; GSE39001 contained 55 samples, including 43 tumor samples and 12 normal samples; GSE46857 contained 29 samples, including 25 tumor samples and 4 normal samples; GSE63514 contained 52 samples, including 28 tumor samples and 24 normal samples; and GSE63678 contained 10 samples, including 5 tumor samples and 5 normal samples. In total, the GEO datasets included 166 samples, comprising 111 tumor samples and 55 normal samples. The information regarding the GEO datasets and sequencing platforms used in this study is presented in Table 1. The TCGA dataset was obtained from the Genomic Data Commons portal (National Cancer Institute, Bethesda, MD, USA; https://portal.gdc.cancer.gov/) and included expression data for 19,938 genes from 304 CC samples and 3 normal cervical tissue samples.

### 2.2. Data Preprocessing

Expression data from the five GEO microarray datasets were preprocessed in R (v4.4.1) using the limma (v3.62.2)package for background correction and normalization. The corrected expression matrices were subsequently merged into a single dataset. To minimize inter-dataset batch effects, the ComBat function in the sva (v3.54.0) package was applied according to batch information. For genes represented by multiple rows in the batch-corrected matrix, the mean expression value was calculated and retained as the representative expression level. Genes with zero expression across all samples were removed. After preprocessing, the merged GEO dataset contained expression data for 3472 genes.

Batch-associated variation before and after ComBat correction was quantified using two complementary PERMANOVA approaches, with principal component analysis (PCA) providing a visual assessment of sample distributions.

First, an overall PERMANOVA test was used to evaluate the total variance explained by the full model, which included both batch and disease group. Before correction, the full model explained 93.32% of the total variance (*R*^2^ = 0.9332, *p* = 0.001). After ComBat correction, the proportion of variance explained by the full model decreased to 4.55% (*R^2^* = 0.0455, *p* = 0.001).

Second, sequential PERMANOVA (with the parameter setting by = “terms”) was used to partition the variance attributable to batch and disease group, with batch entered first in the model. Before correction, batch accounted for 92.96% of the total variance (*R*^2^ = 0.9296, *p* = 0.001), whereas disease group accounted for only 0.36% (*R*^2^ = 0.0036, *p* = 0.001), indicating a pronounced batch effect. After ComBat correction, the variance attributable to batch decreased to 0.018% (*R*^2^ = 0.00018, *p* = 1.000), indicating a substantial reduction in batch-associated variation. The biological signal associated with disease group remained detectable, accounting for 4.53% of the total variance (*R*^2^ = 0.0453, *p* = 0.001).

**Figure 1:**
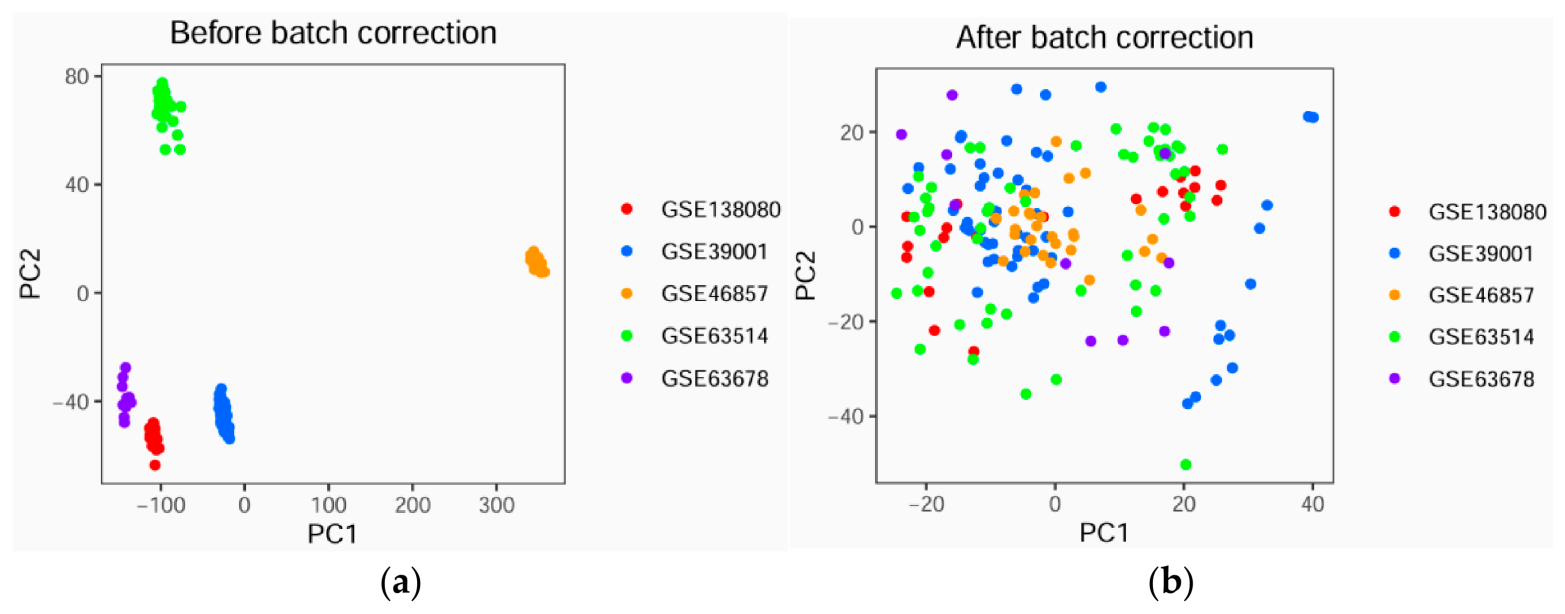
PCA plots of the GEO datasets before and after batch-effect correction. (**a**) PCA plot before batch correction. (**b**) PCA plot after batch correction.

PCA visualization further supported these findings. As shown in Figure 1a, the samples exhibited clear batch separation before correction. In contrast, after correction, the samples were more evenly distributed across batches, indicating that the batch effects were substantially reduced (Figure 1b).

For the TCGA dataset, genes represented by multiple rows were averaged so that only one row per gene was retained. Genes with zero expression across all samples were then removed before subsequent analyses.

### 2.3. Differential Expression Analysis

**Figure 2:**
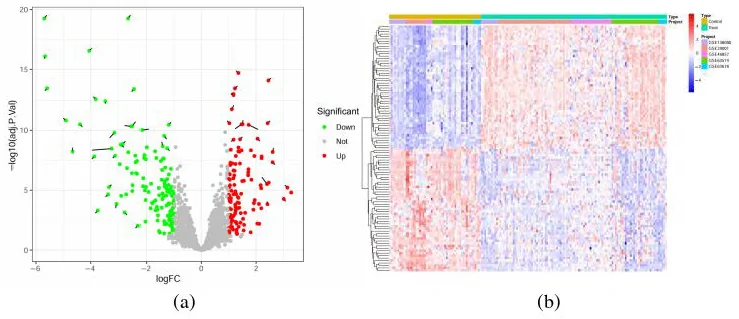
Differential gene expression analysis of cervical cancer and normal samples in the GEO datasets. (**a**) Volcano plot of differentially expressed genes. Red indicates significantly upregulated genes in cervical cancer relative to normal tissues, whereas green indicates significantly downregulated genes. (**b**) Heatmap of differentially expressed genes. Blue indicates relatively low expression, and red indicates relatively high expression.

**Figure 3:**
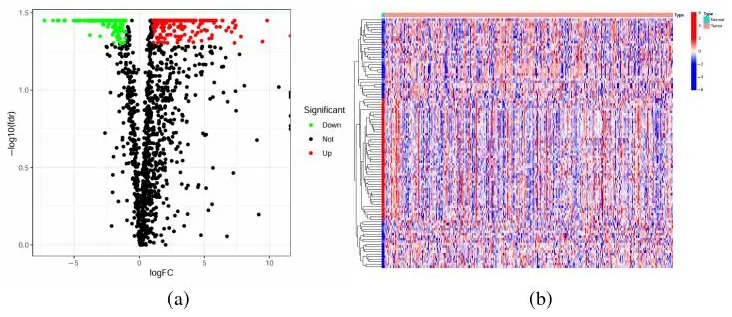
Differential gene expression analysis of cervical cancer and normal samples in the TCGA dataset. (**a**) Volcano plot of differentially expressed genes. Red indicates significantly upregulated genes in cervical cancer relative to normal tissues, whereas green indicates significantly downregulated genes. (**b**) Heatmap of differentially expressed genes. Blue indicates relatively low expression, and red indicates relatively high expression.

**Figure 4:**
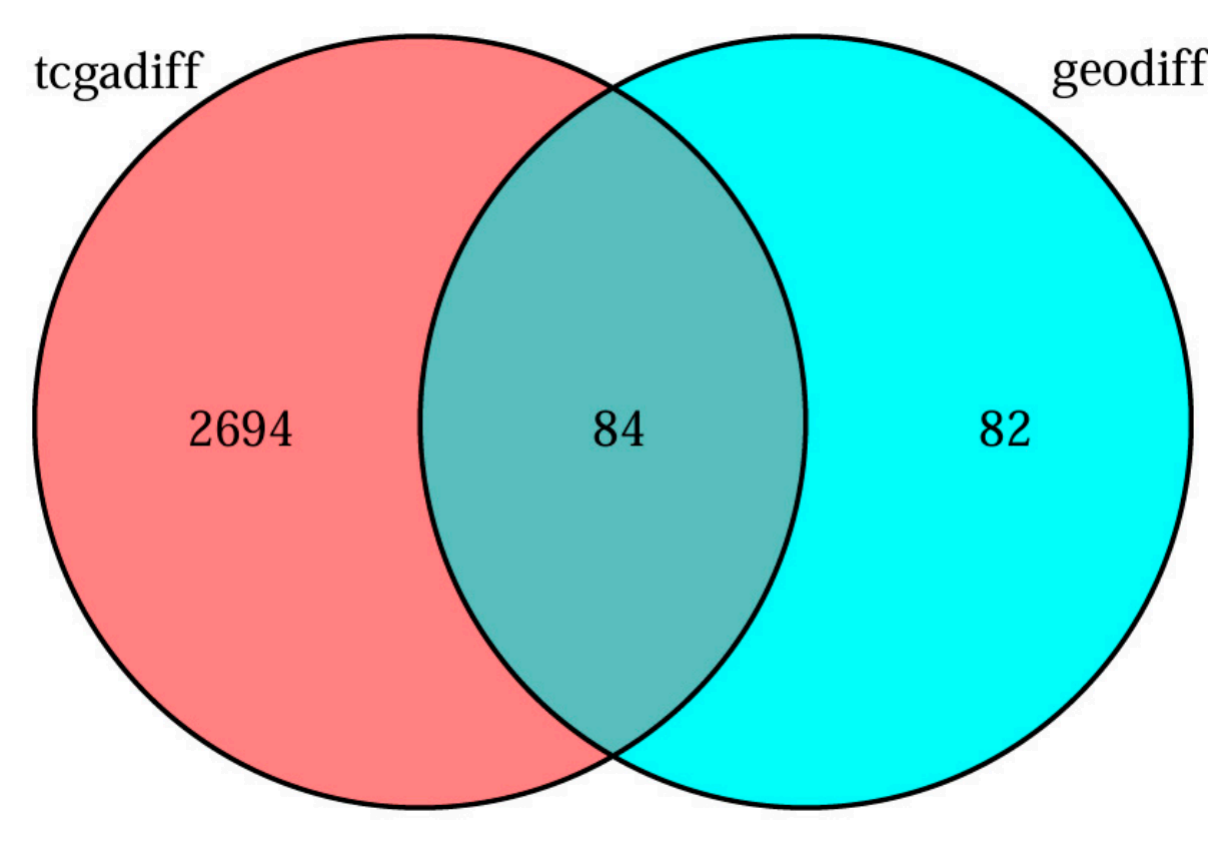
Intersection of DEGs from GEO datasets and TCGA datasets.

Differential expression analysis was performed in R using the limma package. Genes satisfying the criteria of adjusted *p* value < 0.05 and |logFC| > 1 were defined as differentially expressed genes (DEGs). Based on the merged GEO datasets (GSE138080, GSE39001, GSE46857, GSE63514, and GSE63678), 166 significant DEGs were identified (Figure 2a,b). Using the TCGA dataset, 2778 DEGs were detected (Figure 3a,b). The intersection of the GEO-derived and TCGA-derived DEG sets yielded 84 overlapping DEGs, which were retained for subsequent analyses (Figure 4).The Ensembl IDs of the 84 differentially expressed genes are listed in Supplementary Table S1.

### 2.4. Gene Sequence Retrieval and Alignment Design

The overlapping DEGs identified from the GEO and TCGA datasets were used as the target genes for subsequent sequence analysis in order to investigate the positional characteristics of complementary regions between introns and mRNAs. Gene sequence information was retrieved from the Ensembl genome database (http://www.ensembl.org). Briefly, the selected DEGs were first matched to their corresponding Ensembl identifiers, after which the associated gene sequences were downloaded from Ensembl.

During sequence preprocessing, non-coding genes and pseudogenes were excluded. Sequences lacking intron structures were removed, and sequences containing a high proportion of ambiguous nucleotides or repetitive regions were further filtered out to improve the reliability of downstream analyses. After quality control, 84 genes were retained, comprising 486 intron sequences and 84 mRNA sequences.

Based on the processed sequence dataset, three categories of alignment analyses were conducted: (1) alignment between intron sequences and full-length mRNA sequences, including GC-content-stratified analyses; (2) alignment between intron sequences and mRNA sequences in the translation initiation region, including GC-content-stratified analyses; and (3) alignment between intron sequences and mRNA sequences in the translation termination region, including GC-content-stratified analyses.

### 2.5. Construction of the Human Chromosome 1 Background Dataset

To provide a background reference for comparison with cervical cancer-associated differentially expressed genes, genomic gene sequences from human chromosome 1 were used to construct a background dataset. The chromosome 1 gene sequences and their corresponding mRNA and intron sequences were obtained from the Ensembl genome database. Non-coding genes, pseudogenes, genes lacking intron structures, and sequences containing a high proportion of ambiguous nucleotides or repetitive regions were excluded using the same preprocessing criteria applied to the cervical cancer DEG dataset.

The retained chromosome 1 sequences were analyzed using the same Smith–Waterman local alignment procedure and parameter settings described below. For each gene, intron sequences were reverse-complemented and locally aligned with the corresponding mRNA sequence to identify the best-matching region. Matching-frequency distributions were then evaluated for full-length mRNA sequences and for regions surrounding the translation initiation and termination sites. GC-content stratification was performed using the same thresholds applied to the cervical cancer DEG dataset, with fragments containing GC content ≤ 30% assigned to the low-GC group and those containing GC content ≥ 50% assigned to the high-GC group. The resulting chromosome 1 matching patterns were used as a background reference for subsequent comparison with the cervical cancer DEG dataset.

### 2.6. Alignment Algorithm

Sequence alignment was performed using the Smith–Waterman algorithm. Among classical dynamic programming methods, the Needleman–Wunsch algorithm is mainly used for global alignment, whereas the Smith–Waterman algorithm is designed for local alignment and is therefore more suitable for identifying highly similar local regions between biological sequences [27,28,29]. Because the present study focused on detecting local complementary regions between introns and their corresponding mRNAs, the Smith–Waterman algorithm was selected.

For each target gene, the intron sequence was first converted into its reverse-complement sequence and then aligned against the corresponding mRNA sequence. Local alignment was performed using the EDNAFULL scoring matrix, with a gap opening penalty of 50.0 and a gap extension penalty of 5.0. For each sequence pair, the highest-scoring local alignment was defined as the best-matching region, and the start and end positions of the aligned fragment were recorded for subsequent analyses.

**Figure 5:**
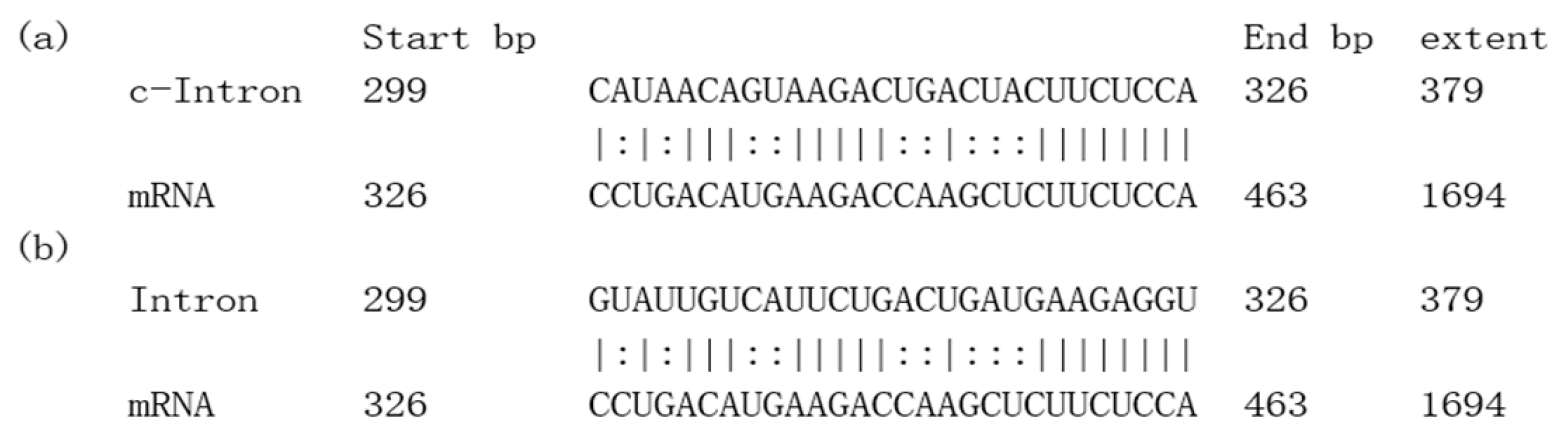
Representative Smith–Waterman local alignment between an intronic sequence and its cognate mRNA. (**a**) Similarity alignment. (**b**) Refined best-matching region. c-Intron denotes the optimal matching fragment of the reverse-complement intron sequence identified by Smith–Waterman local alignment.

The Smith–Waterman algorithm identifies the optimal local alignment through dynamic programming by evaluating matches, mismatches, and gaps at each matrix position. Traceback from the highest-scoring cell to the first zero-valued cell yields the optimal local matching fragment [30,31]. In this study, this procedure was used to determine the most complementary local region between each intron sequence and its cognate mRNA sequence. The resulting aligned fragment was treated as the strongest candidate matching region for downstream analysis, following the alignment strategy reported previously. A representative example of the alignment is shown in Figure 5.

### 2.7. Analysis Steps of Matching Frequency

To characterize the matching patterns between intron sequences and their corresponding mRNA sequences, a five-step analytical procedure was used.

Step 1: Sequence alignment. For each target gene, the intron sequence was aligned with its corresponding mRNA sequence using the Smith–Waterman local alignment algorithm in order to identify the best-matching region and determine its position on the mRNA sequence.

Step 2: Binary encoding of alignment results. After alignment, nucleotide positions within the best-matching region were assigned a value of 1, whereas all remaining positions were assigned a value of 0. This generated a binary sequence that represented the positional distribution of intron–mRNA matching.

Step 3: Sequence standardization. Because mRNA sequences differed in length, direct comparison of matching patterns across genes could be affected by sequence-length heterogeneity. To improve comparability, all mRNA sequences were normalized to a standardized length of 100 nucleotides. The corresponding binary alignment sequences were scaled proportionally in the same manner.

Step 4: Calculation of matching frequency. For each standardized position, the number of sequences assigned a value of 1 was counted across all samples and divided by the total number of samples. This ratio was defined as the matching frequency, which reflects the relative tendency of a given position to participate in intron–mRNA matching.

Step 5: Positional analysis of matching patterns. The distribution of matching frequencies along the full-length standardized mRNA sequences was then analyzed to characterize the overall positional features of intron–mRNA matching.

#### 2.7.1. Standardization of Sequence Lengths

All target sequences were normalized to a standardized length of 100 nucleotides. The specific procedure for sequence-length standardization was as follows:

(1) $$n_{ij}=\left\{\begin{array}{l} {}[100\times N_{ij}/L_{i}]\;[100\times N_{ij}/L_{i}]\;\mathrm{is}\;\mathrm{integer}\\ {}[100\times N_{ij}/L_{i}]+1\;[100\times N_{ij}/L_{i}]\;\mathrm{is}\;\mathrm{non}\text{-}\mathrm{integer} \end{array}\right.$$

In the sequence-length standardization model, *N_ij_* denotes the original position of the *j*-*th* nucleotide in the *i*-*th* sequence, and *L_i_* denotes the original length of sequence *i*. After normalization to a standardized length of 100 nucleotides, *n_ij_* represents the relative position of the *j*-*th* nucleotide in sequence *i*. The brackets in the formula denote the floor function, which rounds a value down to the nearest integer less than or equal to that value. This transformation reduces sequence-length heterogeneity among samples and allows matching features to be compared on a common positional scale.

#### 2.7.2. Matching Scoring Function

For a set of *m* sequences normalized to 100 nucleotides, *N_is_* and *N_ie_* denote the start and end positions of the best-matching region in sequence *i* (*i* = 1, 2, …, m). For each standardized position *j* (*j* = 1, 2, …, 100), the matching status was represented by a binary indicator function *f_ij_*:

(2) $$f_{ij}=\left\{\begin{array}{l} 1\;N_{is}\leq j\leq N_{ie}\\ 0\;j<N_{is}\;or\;j>N_{ie} \end{array}\right.$$

Here, *f_ij_* = 1 indicates that position *j* belongs to the best-matching region of sequence *i*, whereas *f_ij_* = 0 indicates that it does not.

#### 2.7.3. Matching Frequency Function

For a set of *m* sequences normalized to 100 nucleotides, the matching frequency at each standardized position was calculated by counting the number of sequences with *f_ij_* = 1 at position *j* and dividing this count by the total number of sequences *m*. Accordingly, the matching frequency function *F_j_* was defined as

(3) $$F=\frac{1}{m}\sum_{i=1}^{m}f_{ij}$$

Here, *F* represents the matching frequency at the *j*-*th* standardized position. A higher value indicates that this position is more frequently involved in intron–mRNA matching across sequences, whereas a lower value indicates a lower matching frequency.

## 3. Results

### 3.1. Distribution of Matching Frequencies Along mRNA Sequences Under Different GC Content Levels

To examine the positional distribution of intron–mRNA matching along reference mRNA sequences, all target mRNAs were normalized to a standardized length of 100 nucleotides, and the matching frequency function was calculated for each standardized position. To further evaluate the association with sequence composition, the predicted optimal complementary fragments were divided into two groups according to GC content: fragments with GC content ≤ 30% were assigned to the low-GC group, whereas fragments with GC content ≥ 50% were assigned to the high-GC group. In this study, GC contents of 30% and 50% were used as cutoffs to define the low- and high-GC groups, respectively, approximately corresponding to the 25th and 75th percentiles of the distribution of overall mRNA GC content in our dataset. Based on this distribution, fragments with GC contents ≤30% and ≥50% were selected for comparison to establish AU-rich and relatively GC-rich groups with clearly distinct nucleotide compositions. Previous studies provide a biological rationale for comparing groups defined by GC content. First, the thermodynamic stability of RNA duplexes is jointly influenced by base pairing, nearest-neighbor base stacking, and helix-terminal sequences, indicating that both nucleotide composition and sequence arrangement can affect RNA structural stability [32,33]. Second, RNAs differing in GC content may be subject to distinct protein recognition and post-transcriptional regulation. RBM33 directly recognizes GC-rich elements in transcripts and promotes their nuclear export [34]. Under DNA damage conditions, TIRR-bound mRNAs are significantly enriched for transcripts with lower GC contents, and TIRR contributes to the nuclear export and cytoplasmic P-body localization of a subset of its targets [35,36]. Furthermore, the effects of RNA structure on translation depend on its position and folding context; for example, stable helices near translation initiation sites can reduce translation efficiency [37]. In human cells, AU-rich and GC-rich mRNAs exhibit differences in translation, cytoplasmic storage, and degradation regulation, and some experiments have reported higher protein output from GC-rich mRNAs [38]. The effects of GC content on translation also depend on the transcript region and structural context; for example, stable secondary structures in the 5′UTR may inhibit translation initiation [39]. Collectively, these findings provide a biological context for comparing RNA structural and regulatory features across groups with distinct GC compositions. Accordingly, the 30% and 50% cutoffs were used as operational thresholds to define low- and high-GC subgroups with clearly distinct compositions, rather than as critical points marking specific functional changes. The same thresholds were applied to the best-matching fragments identified across the full mRNA and within the translation initiation and termination regions to maintain consistent group definitions and facilitate comparisons of GC-associated features across regions.

**Figure 6:**
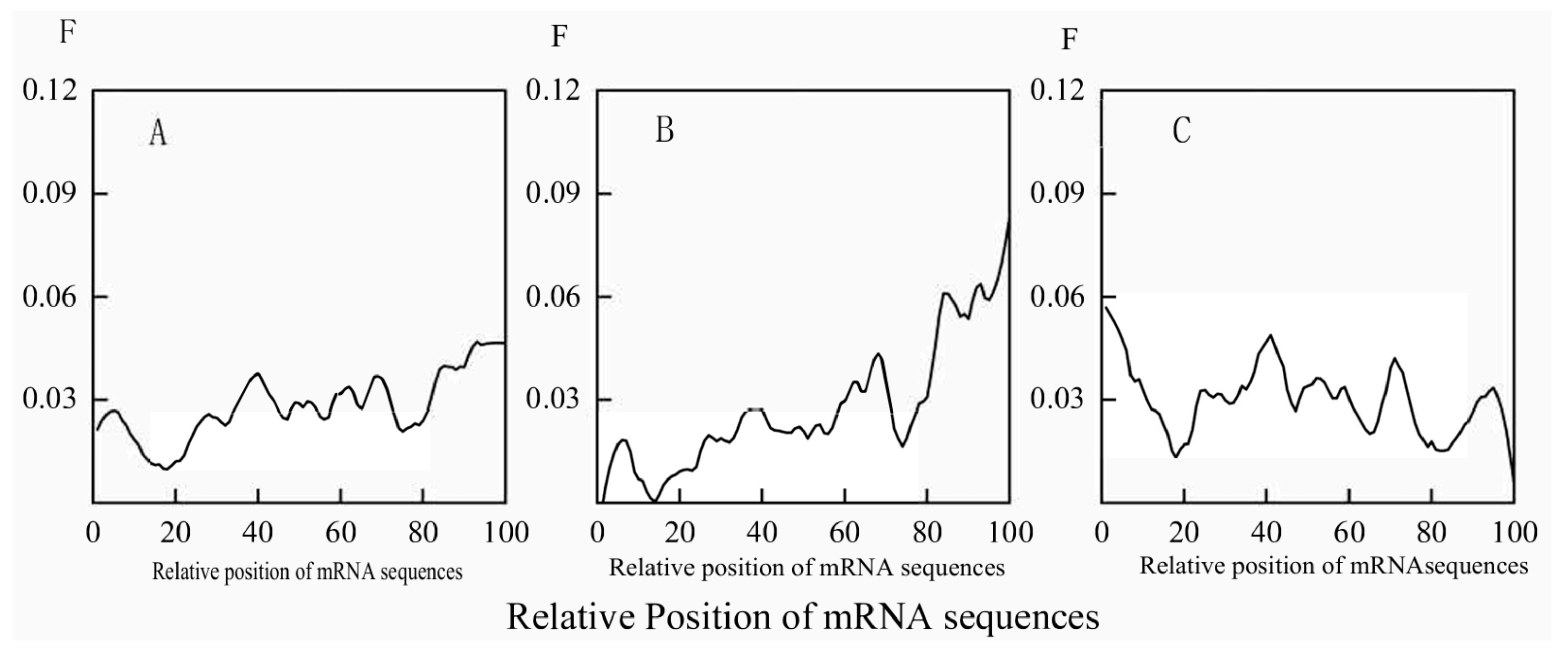
Distribution of matching frequencies between mRNAs of cervical cancer DEGs and their corresponding intron sequences under different GC-content conditions. (**A**) Overall matching-frequency distribution. (**B**) Matching-frequency distribution of low-GC fragments. (**C**) Matching-frequency distribution of high-GC fragments.

In the overall distribution (Figure 6A), two pronounced local minima were observed at approximately 20 nt and 80 nt along the standardized mRNA sequences. Based on the positional mapping used in this study, these regions approximately corresponded to the 5′ untranslated region (5′ UTR) and the 3′ untranslated region (3′ UTR), respectively.

Distinct features of predicted sequence complementarity were observed after GC-content stratification. In the low-GC group (Figure 6B), the matching-frequency profile showed a prominent peak in the 3′ UTR region, indicating that low-GC best-matching fragments were more frequently associated with this region. In contrast, in the high-GC group (Figure 6C), the frequency profile of predicted matching fragments exhibited stronger enrichment in the 5′ UTR region. These results reveal that the positional distribution of computationally predicted intron–mRNA matching differs under different GC-content conditions.

Collectively, these results describe differences in the positional distributions of predicted intron–mRNA matches along normalized full-length mRNAs between the GC-stratified fragment groups.

### 3.2. Distribution of Matching Frequencies in Translation Initiation Regions Under Different GC Content

To examine positional differences in intron–mRNA matching around the translation initiation site, an analysis coordinate system centered on the start codon (AUG) was constructed. The AUG codon was defined as position 0, and the analysis window included 30 nucleotides upstream and 60 nucleotides downstream of this site. This window was selected to capture matching features around the translation initiation region while reducing potential boundary effects.

**Figure 7:**
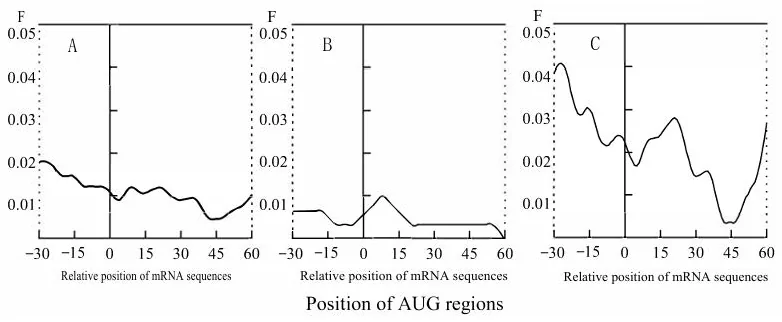
Distribution of matching frequencies in translation initiation regions of cervical cancer DEGs under different GC-content conditions. (**A**) Overall matching-frequency distribution. (**B**) Matching-frequency distribution of low-GC fragments. (**C**) Matching-frequency distribution of high-GC fragments.

In the overall distribution (Figure 7A), matching frequencies were generally higher upstream of the AUG codon than downstream, indicating that computationally predicted intron–mRNA matching occurred more frequently in the upstream region.

After GC-content stratification, distinct regional features were observed. In the low-GC group (Figure 7B), the matching-frequency profile showed a clear local peak near the start codon, whereas the remaining positions were relatively stable. In the high-GC group (Figure 7C), matching frequencies were markedly higher upstream of the AUG codon than downstream, and the downstream region showed more pronounced fluctuations after the start codon was crossed.

Overall, these results indicate that the translation initiation region exhibits clear positional heterogeneity in computationally predicted intron–mRNA matching under different GC-content conditions. Low-GC fragments were characterized by a local enrichment near the start codon, whereas high-GC fragments showed a stronger upstream tendency relative to the downstream region.

### 3.3. Frequency Distribution of Translation Termination Site Matching Across Different GC Content Levels

To examine positional differences in intron–mRNA matching around the translation termination site, an analysis coordinate system centered on the stop codon (UAA, UAG, UGA) was constructed. The stop codon was defined as position 0, and the analysis window included 30 nucleotides immediately upstream of the stop codon, the complete stop codon, and 90 nucleotides immediately downstream of it. This window was selected to capture matching features around the translation termination region while reducing potential boundary effects. Samples were further divided into low-GC and high-GC groups as described above.

**Figure 8:**
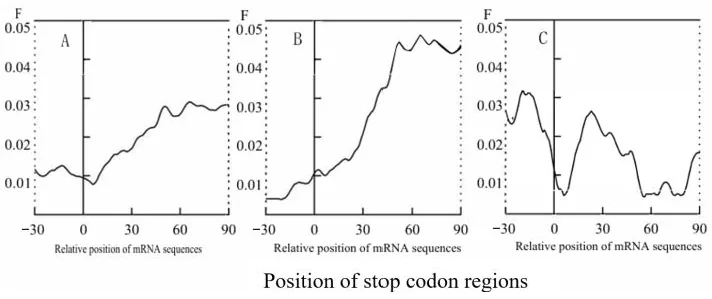
Distribution of matching frequencies in translation termination regions of cervical cancer DEGs under different GC-content conditions. (**A**) Overall matching-frequency distribution. (**B**) Matching-frequency distribution of low-GC fragments. (**C**) Matching-frequency distribution of high-GC fragments.

In the overall distribution (Figure 8A), matching frequency showed an increasing trend from the upstream region toward the downstream region of the stop codon. In general, matching frequencies were higher downstream of the stop codon than upstream, indicating that computationally predicted intron–mRNA matching occurred more frequently in the downstream region.

After GC-content stratification, distinct regional features were observed. In the low-GC group (Figure 8B), matching frequency increased rapidly in the downstream region and reached a prominent peak within the interval immediately following the stop codon, after which it gradually declined. In contrast, in the high-GC group (Figure 8C), the downstream matching frequency was slightly lower than the upstream level, and the profile showed multiple local fluctuations around the stop codon.

The observed termination-region profiles differed between the GC-stratified fragment groups. Low-GC fragments showed a stronger downstream enrichment feature, whereas high-GC fragments displayed a comparatively weaker downstream tendency and a more fluctuating positional profile.

### 3.4. Analysis of Differences in Matching Frequencies

**Figure 9:**
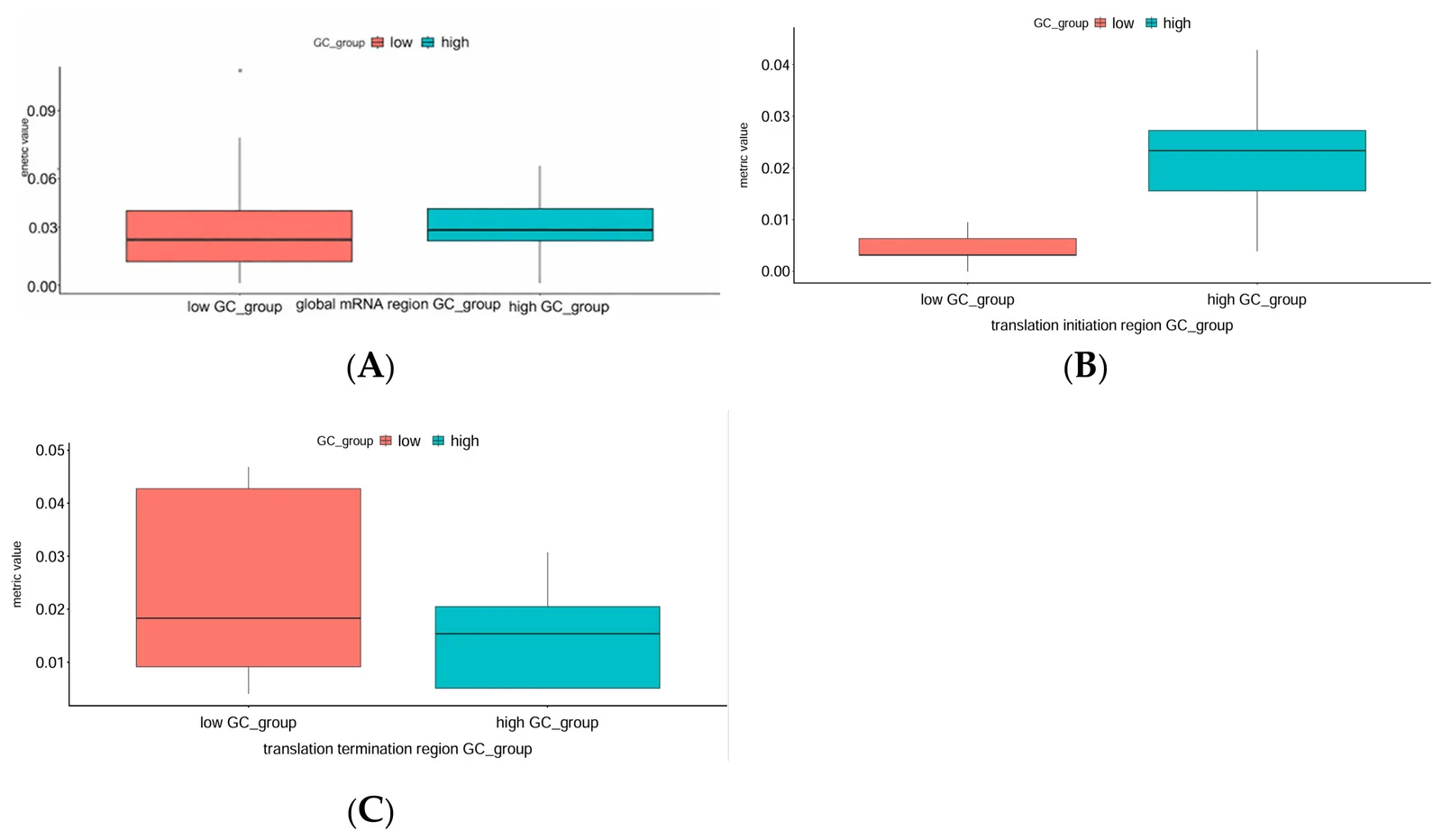
Box plot of predicted matching frequencies between mRNAs of cervical cancer DEGs and their corresponding intron sequences. (**A**) Comparison of full-length mRNA matching frequencies between the low-GC and high-GC groups. (**B**) Comparison of matching frequencies in the translation initiation region between the low-GC and high-GC groups. (**C**) Comparison of matching frequencies in the translation termination region between the low-GC and high-GC groups.

Multiple introns can originate from the same gene, and adjacent positions along an mRNA can be covered by the same aligned fragment. Accordingly, intron-level and position-level observations were not treated as independent replicates, and the matching profiles were summarized descriptively [40]. The observed matching-frequency profiles were compared descriptively between the low- and high-GC fragment groups across the three analyzed regions (Figure 9A–C). Specifically, the matching frequency of full-length mRNA sequences was lower in the low-GC group than in the high-GC group. Similarly, the matching frequency in the translation initiation region was lower in the low-GC group than in the high-GC group. In contrast, the matching frequency in the translation termination region was higher in the low-GC group than in the high-GC group. These descriptive comparisons indicate that the observed differences between GC-content groups vary across the analyzed transcript regions.

As shown in Table 2, comparisons of computationally predicted intron–mRNA matching frequencies between the low-GC and high-GC subgroups revealed regional variation across the three sequence regions analyzed. The reported counts represent the numbers of analyzed positions: 100 normalized positions for full-length mRNAs, 93 nucleotide positions for the translation initiation region, and 123 nucleotide positions for the translation termination region. These counts do not represent numbers of independent genes or introns. The greatest disparity was observed in the translation initiation region, with a very large effect size. Comparisons involving the full-length mRNA sequence and the translation termination region yielded smaller effect sizes, with between-group differences in opposite directions.

## 4. Discussion

Previous studies have shown that computationally predicted complementary sequence matches between introns and their corresponding mRNAs are nonrandomly distributed and may vary with transcript position and sequence composition [9,15,16,17,18]. In the present study, we extended this descriptive analysis to the reference sequences of genes identified as differentially expressed in cervical cancer datasets and compared their matching profiles with those observed in the human chromosome 1 background dataset [15]. Although the two gene sets shared several general trends, the cervical cancer-associated transcript set exhibited distinct regional patterns. The observed matching profiles varied with transcript position and differed between groups stratified by fragment GC content.

Around the translation initiation site, both cervical cancer DEGs and the background group showed higher matching frequencies upstream of the AUG codon than downstream, together with local enrichment of low-GC fragments near the start codon. However, their detailed positional profiles differed. In the chromosome 1 background, the transition around AUG was relatively gradual, whereas cervical cancer DEGs showed a more pronounced feature difference around the AUG boundary, particularly in the high-GC group. These differences indicate that the translation initiation region may represent a sensitive site for characterizing the intrinsic heterogeneity of intron–mRNA matching features.

Differences were also observed around the translation termination site. In both groups, low-GC predicted matching frequencies were relatively low upstream of the stop codon and tended to increase toward the downstream region. However, compared with the background group, cervical cancer DEGs showed lower downstream matching frequencies and greater positional variation for high-GC fragments. Together with the findings for the initiation region, these results indicate that matching fragments are not uniformly distributed along mRNA sequences but vary across functionally distinct transcript regions.

The full-length mRNA analysis further showed that high-GC predicted matching fragments were preferentially distributed toward the 5′ untranslated region, whereas low-GC fragments were more frequently enriched toward the 3′ untranslated region, which is broadly consistent with previous reports [15,18]. Importantly, the magnitude and local distribution of these sequence features differed from those observed in the chromosome 1 background. Because untranslated regions and sequences surrounding translation initiation and termination sites are closely associated with transcript stability, translation efficiency, and post-transcriptional regulation, the observed positional differences may have potential biological relevance.

Several limitations should be considered. This study was based on public transcriptomic datasets and computational sequence analysis, and the identified predicted matching features were not experimentally validated. In addition, the analysis was restricted to overlapping DEGs from GEO and TCGA datasets, and chromosome 1 was used as a background reference rather than a complete normal-genome baseline. Therefore, the observed differences should be interpreted primarily as inherent positional and statistical sequence features of this specific DEG set, rather than as direct evidence of physical interaction or functional regulation. Using a whole-genome protein-coding background, constructing strictly matched random gene sets based on gene length, GC content, intron number and UTR length, and performing multiple permutation tests will be an important optimization and improvement direction for our future follow-up studies. Future studies incorporating broader genomic references, independent clinical cohorts, RNA structural analysis, and functional validation will be needed to determine whether these computational features are associated with RNA regulatory processes and whether they may have potential value as transcript-based molecular features for cervical cancer-related genes.

## 5. Conclusions

This study systematically characterized the matching-frequency features between mRNAs of cervical cancer DEGs and their corresponding intron sequences from the perspectives of sequence position and GC content. The results showed that intron–mRNA matching varied across different functional transcript regions and exhibited positional heterogeneity within this DEG set. High-GC predicted matching fragments were preferentially distributed toward the 5′ untranslated region, whereas low-GC predicted matching fragments were more frequently enriched toward the 3′ untranslated region. Distinct positional patterns were also observed around translation initiation and termination sites. Compared with the chromosome 1 background reference, this set of cervical cancer-associated transcripts showed more pronounced feature differences around the AUG boundary and asymmetric distributions of high- and low-GC fragments in the translation initiation and termination regions. These findings are broadly consistent with previous studies showing that complementary regions between introns and mRNAs are unevenly distributed along transcripts and are influenced by sequence composition.

Based on these exploratory computational findings, future studies may further examine the reproducibility of the observed intron–mRNA matching patterns using composition-preserving sequence controls and independent gene sets. Integration with public transcriptomic datasets and data from independent clinical cohorts may help explore potential associations between these sequence-level features and RNA-processing events, such as intron retention [41,42]. Further computational analyses and experimental studies, potentially combining targeted in vivo RNA structure probing (e.g., DMS-MaPseq [43]) with RNA–RNA interaction mapping (e.g., LIGR-seq [44]), could help characterize local RNA secondary structure and investigate potential RNA–RNA interactions involving selected candidate regions, thereby informing subsequent assessments of the biological relevance of the predicted matching patterns.

## Figures and Tables

**Table 1:** Information on GEO Datasets and Sequencing Platforms Used in This Study.

Dataset	Platform	Relation
GSE138080	GPL4133	https://www.ncbi.nlm.nih.gov/bioproject/PRJNA574467 (accessed on 16 February 2025)
GSE39001	GPL6244	https://www.ncbi.nlm.nih.gov/bioproject/PRJNA169562 (accessed on 16 February 2025)
GSE46857	GPL7025	https://www.ncbi.nlm.nih.gov/bioproject/PRJNA202863 (accessed on 16 February 2025)
GSE63514	GPL570	https://www.ncbi.nlm.nih.gov/bioproject/PRJNA268109 (accessed on 16 February 2025)
GSE63678	GPL571	https://www.ncbi.nlm.nih.gov/bioproject/PRJNA268676 (accessed on 16 February 2025)

**Table 2:** Descriptive comparison between low-GC and high-GC Groups.

Measure	mRNA Sequences	Translation Initiation Region	Translation Termination Region
Low-GC Group number of analyzed Positions: n_1_	n_1_ = 100	n_1_ = 93	n_1_ = 123
High-GC Group number of analyzed Positions: n_2_	n_2_ = 100	n_2_ = 93	n_2_ = 123
Low-GC group sample median	0.02	0	0.02
High-GC group sample median	0.03	0.02	0.02
Cliff’s delta (magnitude)	0.14	0.78	0.21
Probability of Superiority PS	0.44	0.11	0.62

## Data Availability

The datasets analyzed in this study are publicly available in the Gene Expression Omnibus (GEO) under accession numbers GSE138080, GSE39001, GSE46857, GSE63514, and GSE63678, in The Cancer Genome Atlas (TCGA) through the Genomic Data Commons portal, and in the Ensembl genome database. The custom scripts used in this study are available from the corresponding author upon reasonable request.

## Supplementary Materials

The following supporting information can be downloaded at https://www.mdpi.com/article/10.3390/biology15212004/s1.
