# Supplementary material for: Intron–mRNA Matching Patterns in Differentially Expressed Genes in Cervical Cancer
Source: Biology (Basel). 2026 Nov 5;15(21):2004. doi: 10.3390/biology15212004 (PMC13761795; doi:10.3390/biology15212004)
Supplement: Supplementary file 1 [file biology-15-02004-s001.zip › biology-4603914-supplementary.pdf]

Table S1： Ensembl Gene ID

ensembl_gene_id	external_gene_name	ensembl_gene_id	external_gene_name
ENSG00000183036	PCP4	ENSG00000197299	BLM
ENSG00000101955	SRPX	ENSG00000112312	GMNN
ENSG00000196074	SYCP2	ENSG00000089685	BIRC5
ENSG00000179750	APOBEC3B	ENSG00000100526	CDKN3
ENSG00000131831	RAI2	ENSG00000101224	CDC25B
ENSG00000163132	MSX1	ENSG00000138755	CXCL9
ENSG00000157456	CCNB2	ENSG00000169245	CXCL10
ENSG00000145147	SLIT2	ENSG00000240583	AQP1
ENSG00000101057	MYBL2	ENSG00000172037	LAMB2
ENSG00000183580	FBXL7	ENSG00000130208	APOC1
ENSG00000172201	ID4	ENSG00000123975	CKS2
ENSG00000136824	SMC2	ENSG00000196569	LAMA2
ENSG00000134760	DSG1	ENSG00000120885	CLU
ENSG00000106483	SFRP4	ENSG00000072571	HMMR
ENSG00000132646	PCNA	ENSG00000143799	PARP1
ENSG00000100297	MCM5	ENSG00000129009	ISLR
ENSG00000167779	IGFBP6	ENSG00000163918	RFC4
ENSG00000082397	EPB41L3	ENSG00000262406	MMP12
ENSG00000173156	RHOD	ENSG00000142731	PLK4
ENSG00000160213	CSTB	ENSG00000187608	ISG15
ENSG00000164109	MAD2L1	ENSG00000107562	CXCL12
ENSG00000113368	LMNB1	ENSG00000082175	PGR
ENSG00000291560	PCP4	ENSG00000123473	STIL
ENSG00000288508	ISLR	ENSG00000152583	SPARCL1
ENSG00000288364	NDN	ENSG00000112984	KIF20A
ENSG00000087586	AURKA	ENSG00000121552	CSTA
ENSG00000156970	BUB1B	ENSG00000113569	NUP155
ENSG00000176890	TYMS	ENSG00000131153	GINS2
ENSG00000104332	SFRP1	ENSG00000173207	CKS1B
ENSG00000164611	PTTG1	ENSG00000017427	IGF1
ENSG00000069702	TGFBR3	ENSG00000189058	APOD
ENSG00000101868	POLA1	ENSG00000012048	BRCA1
ENSG00000109846	CRYAB	ENSG00000078081	LAMP3
ENSG00000108106	UBE2S	ENSG00000115415	STAT1
ENSG00000145386	CCNA2	ENSG00000149554	CHEK1
ENSG00000117650	NEK2	ENSG00000113810	SMC4
ENSG00000088002	SULT2B1	ENSG00000073111	MCM2
ENSG00000134755	DSC2	ENSG00000108352	RAPGEFL1
ENSG00000182636	NDN	ENSG00000094804	CDC6
ENSG00000046604	DSG2	ENSG00000131747	TOP2A
ENSG00000076382	SPAG5	ENSG00000167900	TK1
ENSG00000097046	CDC7	ENSG00000076003	MCM6
